# Adherence to the planetary health diet index and urinary phytoestrogen excretion: a nationally representative cross-sectional study of US adults

**DOI:** 10.3389/fnut.2026.1915004

**Published:** 2026-10-01

**Authors:** Kyoung-Bok Min, Jin-Young Min

**Affiliations:** 1 Department of Preventive Medicine, Seoul National University College of Medicine, Seoul, Republic of Korea; 2 Integrated Major in Innovative Medical Science, Seoul National University Graduate School, Seoul, Republic of Korea; 3 Institute of Health and Environment, Kongju National University, Gongju-si, Chungcheongnam-do, Republic of Korea

**Keywords:** diet quality, isoflavones, lignans, NHANES, plant-based dietary pattern, sustainable diet

## Abstract

**Background:**

The Planetary Health Diet Index (PHDI) was developed to quantify adherence to dietary patterns that support both human health and environmental sustainability. However, whether higher PHDI adherence is accompanied by measurable differences in diet-derived biochemical exposure remains unclear. We examined the association between PHDI adherence and urinary phytoestrogen excretion among U.S. adults.

**Methods:**

We analyzed cross-sectional data from 5,416 adults participating in the National Health and Nutrition Examination Survey (NHANES) 2003–2010. Dietary intake was assessed using two 24-h dietary recalls, and PHDI scores were calculated according to the EAT–Lancet-based scoring framework. Urinary phytoestrogens, including lignans, isoflavones, and equol, were creatinine-standardized and natural log-transformed. Survey-weighted multivariable linear regression models between PHDI quintiles and urinary phytoestrogen concentrations, using the lowest quintile as the reference. Complementary analyses modeled PHDI continuously per 1-SD higher score. Component-level analyses were conducted with false discovery rate (FDR) correction.

**Results:**

In fully adjusted quintile-based analyses, participants in the highest PHDI quintile had higher urinary concentrations of total phytoestrogens, total isoflavones, and total lignans than those in the lowest quintile, with positive graded trends across quintiles for all three composite measures. In complementary continuous models, each 1-SD higher PHDI was associated with 23.4% higher total phytoestrogens, 17.4% higher total isoflavones, and 24.6% higher total lignans. Similar associations were observed for daidzein, genistein, O-desmethylangolensin, enterodiol, and enterolactone. In contrast, equol—a microbiota-dependent metabolite—showed no association with PHDI. Component-level analyses indicated that soybean/soy foods were most strongly associated with isoflavones, and whole grains with lignans, consistent with their established dietary sources.

**Conclusion:**

Higher PHDI adherence was associated with higher urinary concentrations of selected plant-derived phytoestrogens among U.S. adults. These findings support biological coherence between planetary health-oriented dietary patterns and systemic exposure to plant-derived bioactive compounds. However, urinary phytoestrogens should be interpreted as complementary population-level exposure indicators of plant-forward dietary patterns rather than validated or specific biomarkers of PHDI adherence.

## 1 Introduction

The Planetary Health Diet, proposed by the EAT–Lancet Commission, is a global reference dietary pattern designed to advance human health while simultaneously reducing environmental pressures (1). It promotes a plant-forward intake, including whole grains, legumes, fruits, vegetables, and nuts, while placing strict constraints on red meat and refined sugars (1). The reference diet is not exclusively plant-based; it also includes modest amounts of selected animal-source foods, including fish and seafood, within recommended intake ranges. Accordingly, the PHDI captures adherence to a multidimensional dietary pattern that combines higher intake of plant-derived foods, lower intake of foods recommended for restriction, and intake of several optimum-range components such as fish and seafood (1–3). Recent studies have applied EAT–Lancet-based dietary indices across diverse populations and reported associations with cardiometabolic health, mortality, nutritional adequacy, and environmental impact measures (4–7).

Despite its rapid uptake, a practical challenge persists: adherence is typically quantified via self-reported instruments vulnerable to systematic and random measurement error (8, 9). Such errors may attenuate associations, introduce reporting-related bias, and obscure biologically meaningful variation in dietary exposure. Biological measures derived from urine or blood can complement self-reported dietary assessment by capturing circulating or excreted compounds that reflect diet-related systemic exposure (10, 11). However, such measures should not be interpreted as direct substitutes for dietary intake assessment or as recovery biomarkers unless their quantitative relationship with intake has been clearly established (12, 13). Therefore, biological characterization may help contextualize how dietary indices such as the PHDI relate to measurable biological signals, without serving as formal validation of the index itself.

Phytoestrogens offer a biologically plausible framework for examining diet-related systemic exposure in relation to the PHDI (14). Their principal subclasses, isoflavones and lignans, derive predominantly from plant foods that are central to the plant-forward dimension of the Planetary Health Diet framework. Isoflavones are especially concentrated in soy-based foods, whereas lignans are more broadly derived from fiber-rich plant sources such as whole grains, seeds, fruits, vegetables, and legumes (14, 15). Because soybean/soy foods and non-soy legumes are treated as separate components in the PHDI scoring framework used in this study (2, 3), this distinction is relevant for interpreting component-level associations with urinary phytoestrogen subclasses. Soy foods are major dietary sources of isoflavones, particularly daidzein and genistein, whereas non-soy legumes contribute more broadly to plant protein, fiber, and other phytochemicals but are not the predominant dietary source of urinary isoflavones. In contrast, animal-source optimum-range components such as fish and seafood are not major sources of phytoestrogens and are therefore expected to be less directly reflected in urinary phytoestrogen profiles.

Unlike simple nutrient proxies, urinary phytoestrogen concentrations integrate dietary intake with downstream biological processing. They reflect a complex interplay between intake, absorption, host metabolism, gut microbial biotransformation, conjugation, and renal excretion (16–18). For example, gut microbial metabolism contributes to the conversion of daidzein to equol and plant lignan precursors to enterolignans, and these conversion processes vary substantially across individuals (16, 17, 19). Thus, urinary phytoestrogens should be interpreted as markers of diet-derived systemic exposure that reflect both intake and post-consumption biological processes (11, 18).

This population-level exposure interpretation is consistent with large-scale surveillance platforms such as NHANES, which employ standardized methods to measure urinary isoflavones and enterolignans (20). Although these metabolites can reflect intake of phytoestrogen-containing foods at the population level (21), their relationship with the PHDI remains largely unexplored. Examining urinary phytoestrogen profiles alongside the PHDI may help determine whether adherence to a planetary health–oriented dietary pattern is accompanied by higher systemic exposure to phytoestrogens. Such associations are best interpreted as population-level diet–exposure relationships, rather than as evidence that urinary phytoestrogens function as quantitative biomarkers or specific biomarkers of individual PHDI adherence.

This study aimed to characterize how adherence to the PHDI relates to urinary phytoestrogen concentrations in a nationally representative U.S. population. Specifically, we examined the dose–response association between PHDI scores and urinary phytoestrogen concentrations and evaluated which PHDI components contribute most to observed associations.

## 2 Methods

### 2.1 Study population

We used data from the NHANES 2003–2010, a continuous series of cross-sectional surveys designed to assess the health and nutritional status of the noninstitutionalized US civilian population. NHANES employs a complex, multistage, probability sampling design involving household interviews and physical examinations conducted in Mobile Examination Center (MEC). During each survey year, approximately 15 counties, or primary sampling units, are selected from counties across the United States. However, detailed geographic identifiers, including state, county, and region, are not publicly released in NHANES data files to protect participant confidentiality. Therefore, the geographic distribution of the final analytic sample could not be reported at the state, county, or regional level. The survey protocols were reviewed and approved by the National Center for Health Statistics Research Ethics Review Board, and all participants provided written informed consent prior to data collection. Among the 41,156 individuals initially identified across four survey cycles (2003–2010), we applied sequential exclusion criteria to derive the final analytical sample. A total of 19,214 nonpregnant and nonlactating participants aged 20 years or older who provided two complete days of valid 24-h dietary recall data were eligible after excluding participants with implausible energy intake. Implausible energy intake was defined as a mean daily energy intake of <500 or >8,000 kcal/day based on the average of the two recall days, consistent with prior NHANES-based PHDI research (22). From this eligible pool, we further excluded 13,126 individuals with missing data for all six primary urinary phytoestrogen biomarkers. Additionally, 672 participants were excluded due to missing data on essential covariates, including sociodemographic characteristics, lifestyle factors, or clinical health status. Consequently, a final sample of 5,416 participants was included in the present analysis.

### 2.2 Quantification of urinary phytoestrogen

Spot urine specimens were collected from participants during the MEC visit according to standardized NHANES protocols. The first 24-h dietary recall was also conducted in person during the MEC visit, whereas the second 24-h recall was administered by telephone 3 to 10 days later. Thus, spot urine collection occurred during the same MEC visit as the Day-1 dietary recall, but did not coincide with the Day-2 recall. NHANES measured urinary phytoestrogens in spot urine samples rather than 24-h urine collections; therefore, total 24-h urinary excretion could not be evaluated in this study. No phytoestrogen-specific dietary restriction or timing protocol was available in the public-use NHANES documentation. Therefore, urinary phytoestrogen concentrations should be interpreted as biomarkers of recent diet-related exposure rather than exact temporal matches to the averaged two-day dietary intake used to calculate the PHDI.

Urinary phytoestrogens in NHANES were measured in a random one-third subsample of participants aged 6 years and older; accordingly, all analyses incorporated the phytoestrogen-specific subsample weights to account for the complex sampling design. Specimens were processed, aliquoted, and stored under frozen conditions until shipment to the National Center for Environmental Health at the Centers for Disease Control and Prevention (CDC) for analysis.

Total concentrations, including free and conjugated forms, of six urinary phytoestrogens were quantified. These included four isoflavones or isoflavones-related compounds—daidzein, genistein, O-desmethylangolensin [O-DMA], and equol—and two enterolignans, enterodiol and enterolactone. O-DMA and equol are microbiota-derived metabolites of daidzein, whereas enterodiol and enterolactone are microbiota-derived metabolites of plant lignan precursors. For the 2003–2004 cycle, high-performance liquid chromatography–tandem mass spectrometry (HPLC-MS/MS) was used, transitioning to HPLC-atmospheric pressure photoionization-tandem mass spectrometry for the 2005–2010 cycles. Analytical consistency across methodologies was confirmed through a crossover validation study (15, 20), demonstrating high concordance (r > 0.99) with a regression slope of approximately 1.0 and an intercept near zero. The limit of detection (LOD) ranged from 0.04 ng/mL for enterodiol to 0.4 ng/mL for daidzein. Inter-assay coefficients of variation for the phytoestrogen analytes ranged from approximately 3.1 to 12%, and bench quality control samples were analyzed concurrently with each batch of study specimens to ensure assay precision throughout the measurement period. Concentrations below the LOD were imputed as the LOD divided by the square root of 2, consistent with prior NHANES phytoestrogen studies.

To account for variations in urine dilution among spot specimens, all individual phytoestrogen concentrations were standardized to urinary creatinine and expressed as nanograms per milligram of creatinine (ng/mg creatinine). Composite exposure biomarkers were subsequently derived by summing the creatinine-standardized concentrations of their constituent analytes: total isoflavones, comprising daidzein, genistein, O-DMA, and equol; total lignans, comprising enterodiol and enterolactone; and total phytoestrogens, defined as the sum of all six metabolites. O-DMA and equol are microbiota-derived metabolites of daidzein, whereas enterodiol and enterolactone are microbiota-derived metabolites of plant lignan precursors. Given the right-skewed distributions of phytoestrogen concentrations, all creatinine-standardized values were natural log–transformed prior to regression analyses (see Statistical analysis).

Composite exposure biomarkers were subsequently derived by summing the creatinine-standardized concentrations of their constituent analytes: total isoflavones, comprising daidzein, genistein, O-DMA, and equol; total lignans, comprising enterodiol and enterolactone; and total phytoestrogens, defined as the sum of all six metabolites. O-DMA and equol are microbiota-derived metabolites of daidzein, whereas enterodiol and enterolactone are microbiota-derived metabolites of plant lignan precursors.

### 2.3 Calculation of the PHDI

Dietary intake data were derived from the NHANES (2003–2010). Intake was assessed using two 24-h dietary recalls administered by trained interviewers via the U.S. Department of Agriculture (USDA) Automated Multiple Pass Method. Participants reported all foods and beverages consumed during the 24 h preceding each interview, with portion sizes estimated using standardized measuring guides. The first recall was conducted face-to-face in the MEC, followed by a second interview conducted via telephone 3 to 10 days later (23). Mean intake from the two recall days was used to improve the stability of dietary intake estimates.

The PHDI was calculated using the dietaryindex R package in R version 4.4.2 (24). We used the NHANES-specific implementation, which links dietary recall data with the USDA Food Patterns Equivalents Database and applies the updated PHDI scoring framework based on the EAT–Lancet reference diet (1, 3). In accordance with the updated PHDI framework described by Bui et al. (3), the PHDI used in this study comprised 15 dietary components and ranged from 0 to 140 points, with higher scores indicating greater adherence to the PHD. Component scores were assigned according to the direction of the PHDI recommendation. Adequacy components included whole grains, whole fruits, non-starchy vegetables, nuts and seeds, non-soy legumes, and soybean/soy foods. Higher intake of these foods, up to the recommended level, corresponded to higher component scores. In the NHANES implementation, legumes were represented by two separate components: non-soy legumes and soybean/soy foods. Each component contributed up to 5 points, collectively contributing a maximum of 10 points. This distinction follows the updated PHDI scoring framework and is relevant to the present analysis because soy foods are major dietary sources of isoflavones, particularly daidzein and genistein, whereas non-soy legumes contribute more broadly to plant protein, fiber, and other phytochemicals.

Optimum-range components included fish/seafood, eggs, dairy, poultry, starchy vegetables, and unsaturated oils. For these components, higher scores reflected intake closer to the recommended range rather than simply higher intake. Components to limit included red and processed meats, saturated and trans fats, and added sugar and fruit juice. These components were reverse-coded, such that lower consumption corresponded to higher component scores. Thus, all component scores contributed positively to the total PHDI score; however, for reverse-coded components, higher scores indicate lower intake and greater adherence to PHDI recommendations. Component scores were assigned proportionally for intakes between the minimum and maximum scoring thresholds, thereby capturing a continuous range of adherence rather than using an all-or-nothing approach. The total PHDI score was calculated by summing all component scores. For statistical analyses, PHDI scores were examined both continuously and by quintiles to evaluate graded associations with urinary phytoestrogen concentrations.

### 2.4 Covariates and subgroup variables

Covariates were selected based prior literature and included demographic characteristics, lifestyle, and dietary factors. Demographic and socioeconomic variables included age (continuous), sex, race/ethnicity (Non-Hispanic White, Non-Hispanic Black, Mexican American/Hispanic, and others), and annual family income (less than $20,000 or $20,000 and more). Lifestyle variables included smoking status (never, former, or current smoker), alcohol drinking (yes/no), and moderate physical activity (yes/no). Total energy intake (kcal/day) was calculated as the mean of the two 24-h dietary recalls and included as a dietary covariate in the primary multivariable models.

Health status variables were used for descriptive and stratified analyses. Obesity was defined as body mass index (BMI) ≥ 30 kg/m^2^. Diabetes, hypertension, and dyslipidemia were defined using laboratory measurements, medication use, and self-reported physician diagnosis to reduce under-ascertainment based on self-report alone. Diabetes was defined as HbA1c ≥ 6.5% or fasting plasma glucose ≥126 mg/dL or self-reported physician diagnosis or current use of antidiabetic medication. Hypertension was defined as mean systolic blood pressure ≥140 mmHg or mean diastolic blood pressure ≥90 mmHg (averaged across available MEC measurements) or current use of antihypertensive medication or self-reported physician diagnosis. Dyslipidemia was defined as total cholesterol ≥240 mg/dL or LDL cholesterol ≥160 mg/dL or HDL cholesterol <40 mg/dL (men) / <50 mg/dL (women) or current use of lipid-lowering medication or self-reported physician diagnosis.

Dietary fiber density was calculated as grams of fiber per 1,000 kcal using the mean of the two 24-h dietary recalls. Because dietary fiber may be closely related to both PHDI adherence and urinary phytoestrogen excretion, it was additionally included in fiber-adjusted sensitivity analyses rather than in the primary model.

### 2.5 Statistical analysis

All analyses accounted for the complex, multistage, probability sampling design of NHANES by incorporating survey weights, strata, and primary sampling units. The 2-year mobile examination center sampling weights were rescaled to ensure the combined 2003–2010 cycles represented the non-institutionalized US civilian population. All statistical analyses were conducted using SAS version 9.4 (SAS Institute, Cary, NC, United States). Survey design was incorporated through the SAS SURVEY procedures. Two-sided *p*-values < 0.05 were considered statistically significant.

Descriptive characteristics were summarized across PHDI quintiles. Continuous variables were presented as mean (standard deviation, SD) or mean (standard error, SE), as appropriate, and categorical variables were presented as *n* (%). Age was summarized descriptively but modeled as a continuous variable in regression analyses to preserve information and improve confounding control.

Urinary phytoestrogen outcomes included six individual analytes — daidzein, genistein, equol, O-DMA, enterodiol, and enterolactone—and three composite measures: total phytoestrogens, total isoflavones, and total lignans. All urinary phytoestrogen concentrations were creatinine-standardized and expressed as ng/mg creatinine. Because these concentrations were right-skewed, they were natural log-transformed before regression analyses. Regression coefficients from log-transformed models were exponentiated and expressed as percent differences using the formula (eβ − 1) × 100. To facilitate interpretation on the original concentration scale, observed arithmetric means and 95% confidence intervals were estimated by PHDI quintile and back-transformed to the original concentration scale. To facilitate interpretation on the original concentration scale, observed arithmetic means and standard errors of urinary phytoestrogen concentrations were also presented across PHDI quintiles.

Survey-weighted linear regression was used to estimate associations between PHDI and log-transformed urinary phytoestrogen concentrations. PHDI was modeled both continuously per 1-SD higher score (1 SD = 14.22 points on the 0–140 scale) and categorically by quintiles, with the lowest quintile (Q1) as the reference group. Model 1 adjusted for age [continuous], sex, race/ethnicity, and annual family income. Model 2 further adjusted for smoking status, alcohol use, moderate physical activity, and total energy intake. Restricted cubic spline analyses were conducted to explore potential non-linear associations and are presented in Supplementary Figure S2.

To evaluate which PHDI components contributed to the observed associations, separate Model 2–adjusted regressions were fitted for each of the 15 prespecified PHDI components in relation to the three composite phytoestrogen outcomes, resulting in 45 component–outcome combinations. Each component score was standardized to a z-score using the survey-weighted mean and SD within the analytic sample before modeling. *β* coefficients represent the difference in ln(phytoestrogen) per 1-SD higher component score. This component-level analysis was based on prespecified PHDI components and did not involve any data-driven clustering algorithm.

To account for multiple comparisons, the false discovery rate (FDR) was controlled using the Benjamini–Hochberg procedure within prespecified families of tests including the nine urinary phytoestrogen outcomes in the continuous PHDI models, the nine *p*-values for linear trend in the PHDI quintile models, and the 45 component–outcome associations in the component-level analyses. FDR-adjusted *p* < 0.05 was considered statistically significant.

Two sensitivity analyses were conducted. First, dietary fiber density, calculated as grams per 1,000 kcal, was added to Model 2 for the three composite phytoestrogen outcomes to assess whether the associations were explained by fiber intake (Table 1). Second, the main analyses were repeated after excluding participants with implausible mean daily energy intake using stricter sex-specific cutoffs: <600 or >3,500 kcal/day for women and <800 or >4,200 kcal/day for men (Supplementary Table S4).

**Table 1:** Characteristics of the study population according to PHDI quintiles.

Characteristics	All population		PHDI quintiles										*p*-value*
			Q1 (lowest) *n* = 1,083		Q2 *n* = 1,083		Q3 *n* = 1,084		Q4 *n* = 1,083		Q5 (highest) *n* = 1,083		
Age, years, mean (SD)	50.19	(18.10)	44.13	(17.27)	47.86	(18.42)	50.94	(17.88)	53.14	(17.91)	54.89	(16.96)	<0.001
Sex, *n* (%)													<0.001
Men	2,746	(50.70)	714	(65.93)	588	(54.29)	523	(48.25)	464	(42.84)	457	(42.20)	
Women	2,670	(49.30)	369	(34.07)	495	(45.71)	561	(51.75)	619	(57.16)	626	(57.80)	
Race/ethnicity, *n* (%)													<0.001
Non-Hispanic White	2,738	(50.55)	502	(46.35)	526	(48.57)	546	(50.37)	558	(51.52)	606	(55.96)	
Non-Hispanic Black	1,082	(19.98)	281	(25.95)	234	(21.61)	216	(19.93)	182	(16.81)	169	(15.60)	
Hispanic	1,375	(25.39)	265	(24.47)	300	(27.70)	293	(27.03)	288	(26.59)	229	(21.14)	
Others	221	(4.08)	35	(3.23)	23	(2.12)	29	(2.68)	55	(5.08)	79	(7.29)	
Annual family income, *n* (%)													<0.001
< $20,000	1,329	(24.54)	303	(27.98)	311	(28.72)	264	(24.35)	256	(23.64)	195	(18.01)	
≥$20,000	4,087	(75.46)	780	(72.02)	772	(71.28)	820	(75.65)	827	(76.36)	888	(81.99)	
Cigarette smoking, *n* (%)													<0.001
Current smoker	1,208	(22.30)	368	(33.98)	301	(27.79)	237	(21.86)	189	(17.45)	113	(10.43)	
Past smoker	1,431	(26.42)	225	(20.78)	243	(22.44)	303	(27.95)	306	(28.25)	354	(32.69)	
Never smoked	2,777	(51.27)	490	(45.24)	539	(49.77)	544	(50.18)	588	(54.29)	616	(56.88)	
Alcohol drinking, *n* (%)													<0.001
Yes	3,922	(72.42)	841	(77.65)	795	(73.41)	775	(71.49)	745	(68.79)	766	(70.73)	
No	1,494	(27.58)	242	(22.35)	288	(26.59)	309	(28.51)	338	(31.21)	317	(29.27)	
Moderate activity, *n* (%)													0.055
Yes	2,454	(45.31)	492	(45.43)	487	(44.97)	488	(45.02)	469	(43.31)	518	(47.83)	
No	2,887	(53.31)	584	(53.92)	582	(53.74)	576	(53.14)	591	(54.57)	554	(51.15)	
Obesity (BMI ≥ 30 kg/m^2^), *n* (%)													
Yes	1999	(36.91)	416	(38.41)	444	(41.00)	416	(38.38)	389	(35.92)	334	(30.84)	<0.001
No	3,417	(63.09)	667	(61.59)	639	(59.00)	668	(61.62)	694	(64.08)	749	(69.16)	
Comorbidities													
Diabetes	731	(13.50)	126	(11.63)	133	(12.28)	154	(14.21)	158	(14.59)	160	(14.77)	0.104
Hypertension	2072	(38.26)	324	(29.92)	409	(37.77)	416	(38.38)	460	(42.47)	463	(42.75)	<0.001
Dyslipidemia	2096	(38.70)	360	(33.24)	382	(35.27)	432	(39.85)	443	(40.90)	479	(44.23)	<0.001
Dietary and nutritional variables, mean (SD)													
Total energy intake(kcal/d)	2057.49	(832.19)	2434.2	(915.92)	2071.3	(820.44)	1978.7	(803.05)	1918.7	(763.94)	1884.6	(726.05)	<0.001
Fiber intake density (g/1000 kcal)	8.11	(3.77)	5.6	(2.39)	6.8	(2.62)	7.8	(2.96)	9.0	(3.52)	11.3	(4.22)	<0.001

PHDI, Planetary Health Diet Index; SD, standard deviation. PHDI quintile cutpoints: Q1 = 11.1–47.8, Q2 = 47.8–55.3, Q3 = 55.3–62.4, Q4 = 62.4–72.0, Q5 = 72.0–120.2. Continuous variables are presented as median (IQR) for skewed distributions and mean (SD) otherwise. ^*^p-values were calculated by treating the median of each PHDI quintile as a continuous term in survey-weighted linear regression models to test for linear associations across categories.

Exploratory stratified analyses were conducted to assess the consistency of associations across sex, age group (20–44, 45–64, and ≥65 years), race/ethnicity, annual family income (<$20,000 and ≥$20,000), obesity status, and cardiometabolic condition. Obesity was defined as body mass index ≥30 kg/m^2^. Any cardiometabolic condition was defined as the presence of diabetes, hypertension, or dyslipidemia. In stratified analyses, PHDI was modeled continuously per 1-SD higher score, and analyses were restricted to the three composite phytoestrogen outcomes. These analyses were considered exploratory and are presented in Supplementary Table S5.

## 3 Results

### 3.1 Sample characteristics

The analytic sample comprised 5,416 NHANES 2003–2010 participants aged ≥20 years with complete data on the PHDI, urinary phytoestrogens, and study covariates (Supplementary Figure S1). Table 2 shows the characteristics of the sample according to quintiles of the PHDI. Across increasing quintiles of the PHDI, participant characteristics differed markedly. The mean (SD) age was 50.2 (18.1) years. Higher PHDI was associated with older age (p-trend < 0.001) and a higher proportion of women (34.07% in Q1 to 57.80% in the highest quintile (Q5); p-trend < 0.001). Race/ethnicity also varied (p-trend < 0.001): the proportion of non-Hispanic White participants increased (46.35 to 55.96%), whereas non-Hispanic Black participants decreased (25.95 to 15.60%); Hispanic participants were slightly less represented in Q5 (24.47 to 21.14%). Higher PHDI was further associated with higher income (≤$20,000: 27.98 to 18.01%; p-trend < 0.001). Lifestyle profiles were generally healthier at higher PHDI levels. Current smoking declined substantially (33.98 to 10.43%; p-trend < 0.001) and obesity was less prevalent (38.41 to 30.84%; p-trend < 0.001), while moderate activity did not differ materially across quintiles (*p* = 0.055). Alcohol drinking showed modest differences across quintiles (p-trend < 0.001). Comorbidity patterns were mixed: diabetes did not vary by PHDI (*p* = 0.104), whereas hypertension and dyslipidemia increased across quintiles (both p-trend < 0.001), consistent with the older age distribution in higher-PHDI groups. Participants in higher PHDI quintiles had higher fiber intake density and lower total energy intake.

A comparison of included and excluded eligible participants is presented in Supplementary Table S1. The excluded group comprised 13,798 eligible participants, primarily those without urinary phytoestrogen measurements. Included and excluded participants were generally similar in most demographic and clinical characteristics, although annual family income, alcohol drinking, moderate physical activity, and fiber intake density differed significantly between groups.

### 3.2 PHDI and phytoestrogen distribution

The PHDI total score in the analytic sample had a mean of 59.83 (SD 14.22; median 58.86, IQR 49.55–69.16). Urinary phytoestrogens were strongly right-skewed; medians (interquartile range, IQR) of the composite measures were 7.11 (IQR, 3.10–14.95) ng/mg creatinine for total phytoestrogens, 0.95 (IQR, 0.40–3.22) for total isoflavones, and 4.47 (IQR, 1.58–10.08) for total lignans. Detailed distributional characteristics of all individual analytes are provided in Supplementary Table S2.

Supplementary Table S3 presents baseline PHDI component scores across PHDI quintiles. Higher PHDI quintiles generally showed higher scores for recommended and optimum-range components, as well as higher reverse-coded scores for components to limit, indicating greater overall adherence to the PHDI framework.

### 3.3 PHDI quintile analyses and dose–response patterns

Table 3 presents the associations between PHDI quintiles and urinary phytoestrogen concentrations. In the fully adjusted Model 2, log-transformed composite urinary phytoestrogen concentrations generally increased across PHDI quintiles, with p-trend < 0.001 for all three composite measures. Compared with the lowest quintile (Q1), participants in the highest quintile (Q5) had higher concentrations of total phytoestrogens (*β* = 0.82, 95% CI: 0.66–0.97), total isoflavones (*β* = 0.59, 95% CI: 0.40–0.75), and total lignans (*β* = 0.84, 95% CI: 0.65–1.04). For individual analytes, positive trends were observed for daidzein, genistein, O-DMA, enterodiol, and enterolactone, with p-trend < 0.001 for each. The strongest associations were generally observed in Q5, including daidzein (*β* = 0.61, 95% CI: 0.41–0.80), genistein (β = 0.62, 95% CI: 0.42–0.81), O-DMA (β = 0.70, 95% CI: 0.39–1.01), enterodiol (β = 0.81, 95% CI: 0.62–1.01), and enterolactone (β = 0.80, 95% CI: 0.57–1.03). In contrast, equol showed no significant association across PHDI quintiles in the adjusted model (p-trend = 0.6702).

**Table 2:** Associations between PHDI quintiles and urinary phytoestrogen concentrations.

Phytoestrogen	PHDI quintile	Crude		Model 1		Model 2		p-trend (model 2)
		β (95% CI)	*p*-value	β (95% CI)	*p*-value	β (95% CI)	*p*-value	
Composite measures								
Total phytoestrogen	Q1	Reference		Reference		Reference		<0.001
	Q2	0.22 (0.06–0.38)	0.0053	0.13 (−0.03–0.29)	0.1115	0.12 (−0.04–0.28)	0.1475	
	Q3	0.44 (0.26–0.62)	<0.0001	0.30 (0.12–0.48)	0.001	0.29 (0.12–0.45)	0.0016	
	Q4	0.68 (0.52–0.84)	<0.0001	0.48 (0.32–0.64)	<0.0001	0.45 (0.31–0.60)	<0.0001	
	Q5	1.08 (0.92–1.24)	<0.0001	0.86 (0.70–1.02)	<0.0001	0.82 (0.66–0.97)	<0.0001	
Total isoflavones	Q1	Reference		Reference		Reference		<0.001
	Q2	0.15 (−0.03–0.33)	0.0829	0.09 (−0.07–0.25)	0.2883	0.10 (−0.06–0.27)	0.2194	
	Q3	0.09 (−0.07–0.25)	0.2687	−0.01 (−0.17–0.15)	0.8999	0.00 (−0.16–0.15)	0.9969	
	Q4	0.44 (0.26–0.62)	<0.0001	0.30 (0.12–0.48)	0.0012	0.29 (0.13–0.45)	0.0011	
	Q5	0.76 (0.56–0.96)	<0.0001	0.60 (0.40–0.80)	<0.0001	0.59 (0.40–0.75)	<0.0001	
Total lignans	Q1	Reference		Reference		Reference		<0.001
	Q2	0.27 (0.07–0.47)	0.0068	0.16 (−0.04–0.36)	0.1275	0.13 (−0.07–0.33)	0.2186	
	Q3	0.60 (0.36–0.84)	<0.0001	0.44 (0.20–0.68)	0.0005	0.41 (0.18–0.64)	0.0009	
	Q4	0.76 (0.54–0.98)	<0.0001	0.53 (0.31–0.75)	<0.0001	0.48 (0.26–0.70)	<0.0001	
	Q5	1.18 (0.98–1.38)	<0.0001	0.92 (0.72–1.12)	<0.0001	0.84 (0.65–1.04)	<0.0001	
Individual analytes								
Daidzein	Q1	Reference		Reference		Reference		<0.001
	Q2	0.13 (−0.04–0.30)	0.1377	0.07 (−0.11–0.25)	0.4396	0.09 (−0.08–0.27)	0.3169	
	Q3	0.10 (−0.07–0.27)	0.2755	0.01 (−0.18–0.19)	0.9482	0.02 (−0.17–0.21)	0.8319	
	Q4	0.41 (0.21–0.61)	0.0002	0.28 (0.09–0.47)	0.0065	0.29 (0.09–0.49)	0.0056	
	Q5	0.74 (0.54–0.94)	<0.0001	0.60 (0.40–0.80)	<0.0001	0.61 (0.41–0.80)	<0.0001	
Genistein	Q1	Reference		Reference		Reference		<0.001
	Q2	0.17 (0.01–0.34)	0.0471	0.12 (−0.05–0.28)	0.1646	0.14 (−0.02–0.31)	0.0989	
	Q3	0.07 (−0.10–0.23)	0.4299	−0.02 (−0.19–0.15)	0.8232	0.00 (−0.17–0.17)	0.9920	
	Q4	0.44 (0.25–0.64)	<0.0001	0.31 (0.12–0.50)	0.0025	0.33 (0.14–0.52)	0.0013	
	Q5	0.75 (0.54–0.96)	<0.0001	0.60 (0.39–0.81)	<0.0001	0.62 (0.42–0.81)	<0.0001	
O-DMA	Q1	Reference		Reference		Reference		<0.001
	Q2	0.17 (−0.10–0.44)	0.2185	0.08 (−0.18–0.35)	0.5406	0.07 (−0.18–0.32)	0.5888	
	Q3	0.23 (−0.01–0.47)	0.0611	0.07 (−0.16–0.31)	0.5580	0.05 (−0.18–0.29)	0.6607	
	Q4	0.65 (0.41–0.88)	<0.0001	0.43 (0.20–0.65)	0.0005	0.38 (0.15–0.61)	0.0018	
	Q5	1.01 (0.70–1.31)	<0.0001	0.78 (0.47–1.08)	<0.0001	0.70 (0.39–1.01)	<0.0001	
Equol	Q1	Reference		Reference		Reference		0.6702
	Q2	0.08 (−0.03–0.20)	0.1590	0.01 (−0.11–0.13)	0.9092	0.02 (−0.10–0.15)	0.7325	
	Q3	0.10 (−0.05–0.25)	0.1779	−0.03 (−0.19–0.12)	0.6550	−0.02 (−0.18–0.14)	0.8033	
	Q4	0.20 (0.04–0.37)	0.0179	0.04 (−0.13–0.20)	0.6654	0.04 (−0.12–0.20)	0.6284	
	Q5	0.28 (0.14–0.43)	0.0003	0.06 (−0.07–0.20)	0.3371	0.07 (−0.07–0.20)	0.3337	
Enterodiol	Q1	Reference		Reference		Reference		<0.001
	Q2	0.33 (0.16–0.50)	0.0003	0.22 (0.05–0.40)	0.0171	0.22 (0.04–0.40)	0.0196	
	Q3	0.54 (0.33–0.75)	<0.0001	0.39 (0.18–0.60)	0.0006	0.39 (0.19–0.60)	0.0005	
	Q4	0.61 (0.42–0.80)	<0.0001	0.42 (0.22–0.62)	0.0001	0.41 (0.21–0.61)	0.0002	
	Q5	1.07 (0.88–1.27)	<0.0001	0.84 (0.65–1.03)	<0.0001	0.81 (0.62–1.01)	<0.0001	
Enterolactone	Q1	Reference		Reference		Reference		<0.001
	Q2	0.22 (0.03–0.42)	0.0316	0.12 (−0.09–0.34)	0.2689	0.09 (−0.13–0.30)	0.4337	
	Q3	0.61 (0.35–0.86)	<0.0001	0.45 (0.19–0.70)	0.0012	0.41 (0.16–0.66)	0.0025	
	Q4	0.78 (0.56–0.99)	<0.0001	0.56 (0.34–0.78)	<0.0001	0.50 (0.28–0.72)	<0.0001	
	Q5	1.13 (0.89–1.37)	<0.0001	0.89 (0.65–1.12)	<0.0001	0.80 (0.57–1.03)	<0.0001	

PHDI quintile cut-points: Q1 = 11.1–47.8, Q2 = 47.8–55.3, Q3 = 55.3–62.4, Q4 = 62.4–72.0, Q5 = 72.0–120.2. β represents the difference in ln(phytoestrogen) compared with Q1 (reference). p-trend was calculated by modeling the survey-weighted median PHDI value of each quintile as a continuous term. All phytoestrogen concentrations are creatinine-adjusted and natural log–transformed. Crude: unadjusted. Model 1: adjusted for age (continuous), sex, race/ethnicity, and annual family income. Model 2: Model 1 + smoking status, alcohol use, moderate physical activity, and total energy intake.

Figure 1 shows the observed arithmetic means of urinary phytoestrogen concentrations across PHDI quintiles on the original concentration scale. Total phytoestrogens, total isoflavones, and total lignans increased progressively across higher PHDI quintiles. Similar descriptive increasing patterns were observed for most individual analytes, although equol was not significantly associated with PHDI in the adjusted regression models.

**Figure 1:**
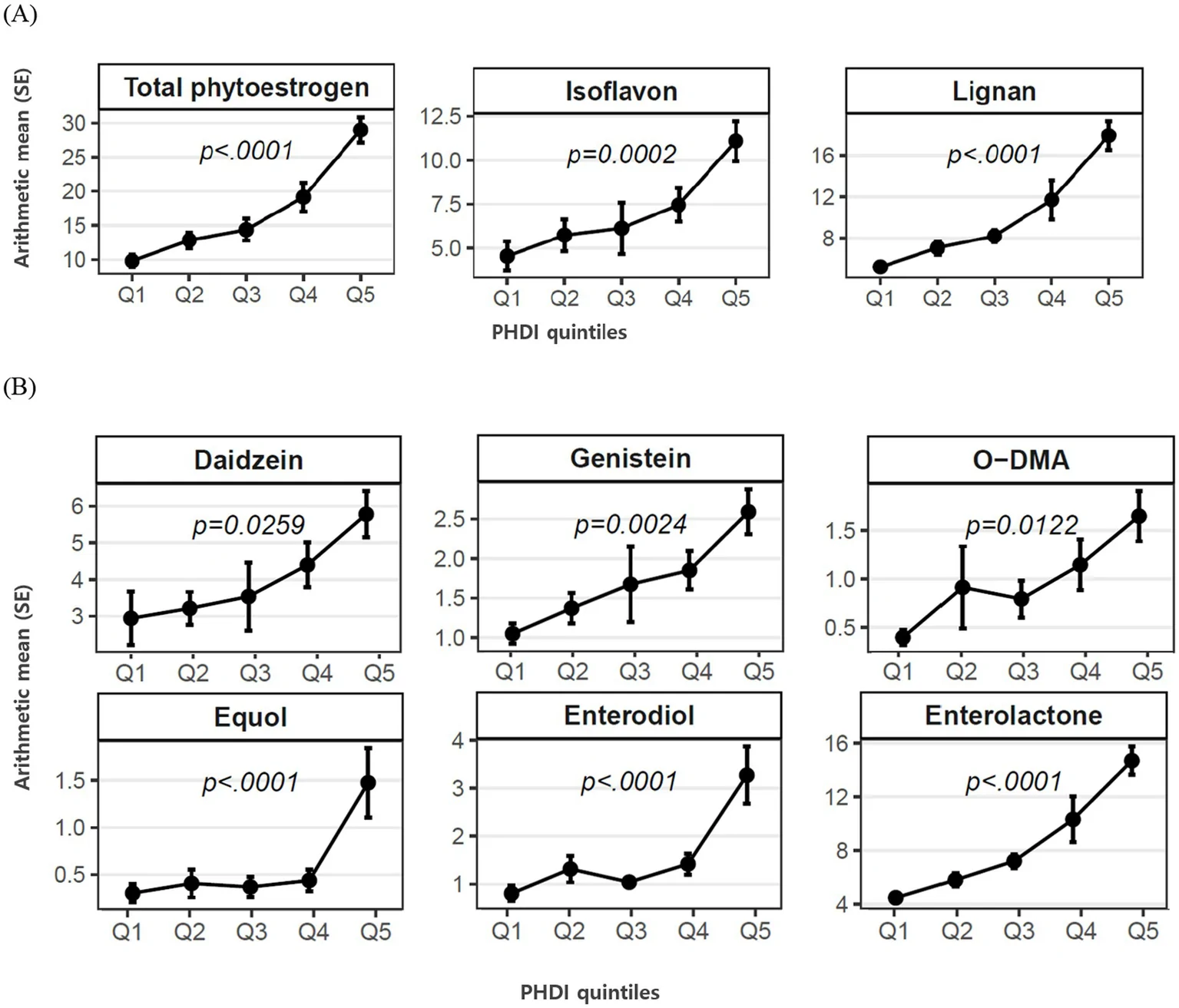
Arithmetic means (SE) of urinary phytoestrogen concentrations (ng/mg creatinine) across quintiles of the PHDI. Observed arithmetic means are presented on the original concentration scale. Error bars represent standard errors. *p*-values shown in each panel are for linear trend across quintiles. PHDI quintile cut points in the analytic sample were Q1 = 11.1–47.8, Q2 = 47.8–55.3, Q3 = 55.3–62.4, Q4 = 62.4–72.0, and Q5 = 72.0–120.2. **(A)** Composite measures. **(B)** Individual analytes.

Restricted cubic spline analyses showed positive dose–response associations between PHDI and composite urinary phytoestrogen outcomes (Supplementary Figure S2). The association appeared approximately linear for total lignans, whereas total isoflavones showed evidence of non-linearity, with a steeper increase at higher PHDI scores.

### 3.4 Continuous PHDI analyses

Table 4 presents associations of the PHDI, modeled per 1-SD higher score, with log-transformed urinary phytoestrogen concentrations. In the fully adjusted Model 2, each 1-SD higher PHDI score, corresponding to a 14.22-point increase, was associated with 23.4% higher total urinary phytoestrogens (*β* = 0.21, 95% CI: 0.18–0.25), 17.4% higher total isoflavones (β = 0.16, 95% CI: 0.12–0.21), and 24.6% higher total lignans (β = 0.22, 95% CI: 0.18–0.26). Associations were broadly consistent across daidzein, genistein, O-DMA, enterodiol, and enterolactone, whereas equol showed no association with PHDI.

**Table 4:** Fiber-adjusted sensitivity analyses of the associations between PHDI and composite urinary phytoestrogen concentrations.

Phytoestrogen	PHDI quintiles (Q1 as reference), β (95% CI), *p*-value								PHDI per 1-SD increase	
	Q2		Q3		Q4		Q5		β (95% CI)	*p*-value
	β (95% CI)	*p*-value	β (95% CI)	*p*-value	β (95% CI)	*p*-value	β (95% CI)	*p*-value		
Total phytoestrogens	0.09 (−0.07–0.25)	0.2858	0.23 (0.06–0.41)	0.0132	0.36 (0.20–0.53)	<0.0001	0.65 (0.46–0.83)	<0.0001	0.18 (0.14–0.22)	<0.001
Total isoflavons	0.09 (−0.08–0.26)	0.2924	−0.02 (−0.20–0.15)	0.7946	0.25 (0.07–0.43)	0.0082	0.51 (0.30–0.73)	<0.0001	0.15 (0.10–0.20)	<0.001
Total lignans	0.10 (−0.11–0.31)	0.3711	0.35 (0.10–0.60)	0.0078	0.38 (0.15–0.61)	0.0024	0.66 (0.40–0.91)	<0.0001	0.18 (0.13–0.23)	<0.001

Values are β coefficients and 95% confidence intervals from survey-weighted linear regression models using natural log-transformed urinary phytoestrogen concentrations. Model 3 was adjusted for age, sex, race/ethnicity, annual family income, smoking status, alcohol use, moderate physical activity, total energy intake, and dietary fiber density. Dietary fiber density was calculated as grams per 1,000 kcal. PHDI quintile analyses used Q1 as the reference group. Continuous PHDI analyses were scaled per 1-SD higher PHDI score.

**Table 3:** Associations between continuous PHDI score and urinary phytoestrogen concentrations.

Phytoestrogen	Crude		Model 1		Model 2		% change
	β (95% CI)	*p*-value	β (95% CI)	*p*-value	β (95% CI)	*p*-value	
Composite measures							
Total phytoestrogens	0.38 (0.33–0.44)	<0.001	0.31 (0.26–0.37)	<0.001	0.21 (0.18–0.25)	<0.001	23.4%
Total isoflavons	0.28 (0.23–0.34)	<0.001	0.23 (0.17–0.28)	<0.001	0.16 (0.12–0.21)	<0.001	17.4%
Total lignans	0.40 (0.34–0.45)	<0.001	0.33 (0.27–0.38)	<0.001	0.22 (0.18–0.26)	<0.001	24.6%
Individual analytes							
Daidzein	0.27 (0.21–0.33)	<0.001	0.23 (0.17–0.28)	<0.001	0.17 (0.12–0.21)	<0.001	18.5%
Genistein	0.28 (0.23–0.34)	<0.001	0.24 (0.19–0.30)	<0.001	0.18 (0.13–0.23)	<0.001	19.7%
O-DMA	0.37 (0.29–0.45)	<0.001	0.28 (0.20–0.37)	<0.001	0.20 (0.13–0.26)	<0.001	22.1%
Equol	0.09 (0.06–0.11)	<0.001	0.01 (−0.04–0.07)	0.3403	0.02 (−0.01–0.05)	0.2529	2.0%
Enterodiol	0.37 (0.31–0.43)	<0.001	0.30 (0.24–0.35)	<0.001	0.21 (0.16–0.25)	<0.001	23.4%
Enterolactone	0.40 (0.31–0.48)	<0.001	0.31 (0.23–0.40)	<0.001	0.21 (0.17–0.26)	<0.001	23.4%

β represents the difference in ln(phytoestrogen) per 1-SD higher PHDI total score (1 SD = 14.22-point increase; analytic sample mean ± SD = 59.83 ± 14.22). Percent change = (e^β^ − 1) × 100. All phytoestrogen concentrations are creatinine-adjusted and natural log–transformed. Crude: unadjusted. Model 1: adjusted for age (continuous), sex, race/ethnicity, and income. Model 2: Model 1 + smoking status, alcohol use, moderate physical activity, and total energy intake. All *p*-values from survey-weighted linear regression. *p*-values from Model 2 were adjusted for multiple testing using the Benjamini–Hochberg false discovery rate (FDR) procedure across the 9 phytoestrogen outcomes; all associations remained statistically significant (FDR-adjusted *p* < 0.001) except for equol (FDR-adjusted *p* = 0.45).

### 3.5 Component-level analyses

Figure 2 shows component-level associations between individual PHDI component scores and composite urinary phytoestrogen outcomes. Among recommended components, soybean/soy foods showed the strongest association with total isoflavones, whereas whole grains were strongly associated with total lignans. Whole fruits, non-starchy vegetables, and nuts and seeds also showed positive associations with composite urinary phytoestrogen outcomes. For components to limit, higher reverse-coded scores for red and processed meat, saturated and trans fats, and added sugar and fruit juice were positively associated with selected outcomes, indicating that lower consumption of these foods was aligned with higher urinary phytoestrogen concentrations.

**Figure 2:**
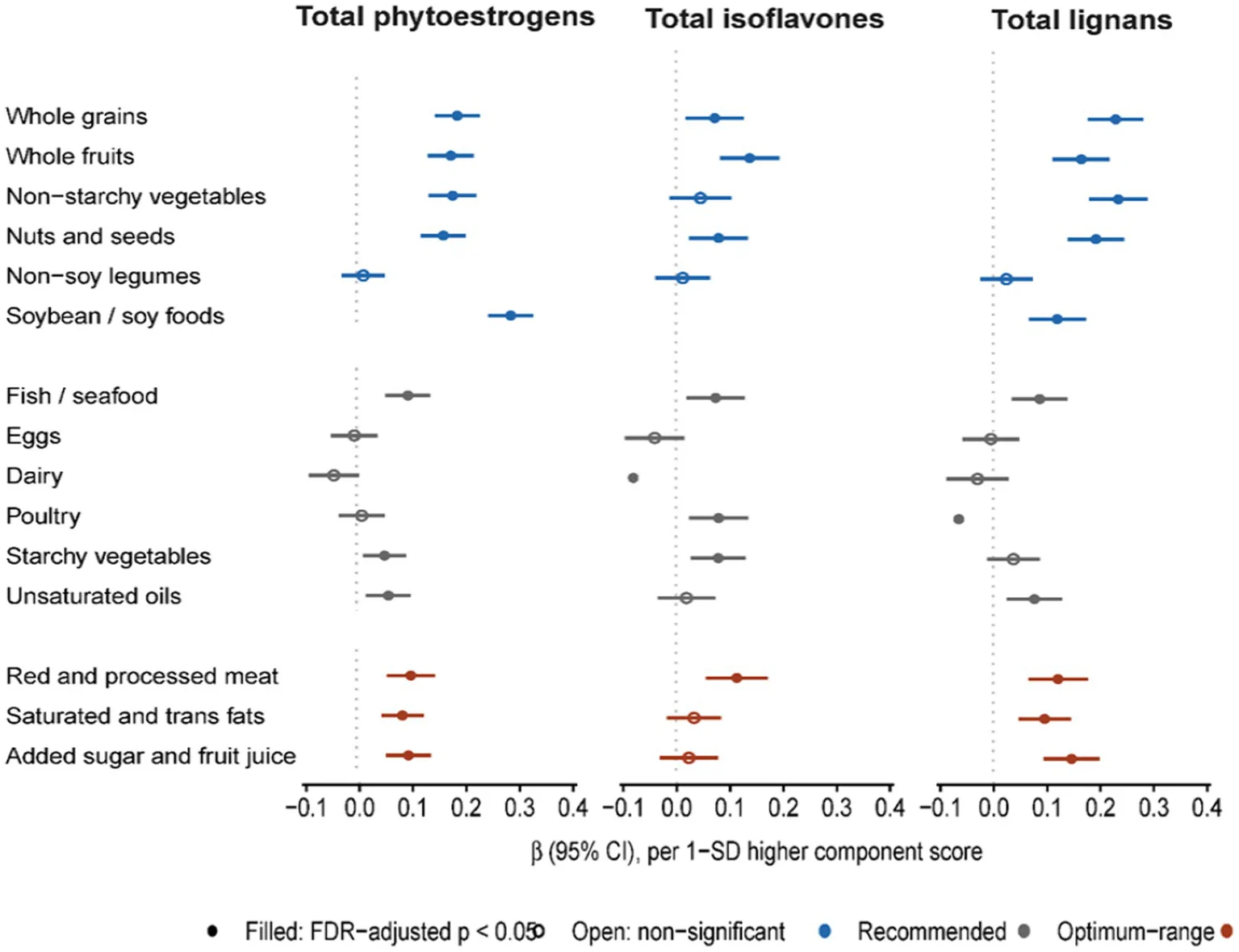
Component-level associations of PHDI scores with composite urinary phytoestrogen concentrations. Each of the 15 PHDI components was standardized to a *z*-score using the survey-weighted mean and standard deviation within the analytic sample and entered as a continuous variable in separate survey-weighted linear regression models. Outcomes were natural *ln*-transformed, creatinine-standardized urinary concentrations of total phytoestrogens (left panel), total isoflavones (middle panel), and total lignans (right panel). All models were adjusted for age, sex, race/ethnicity, annual family income, smoking status, alcohol use, moderate physical activity, and total energy intake. *β* coefficients represent the difference in *ln*-transformed urinary phytoestrogen concentration per 1-*SD* higher PHDI component score, with 95% confidence intervals shown as horizontal error bars. Filled circles indicate associations with Benjamini–Hochberg FDR-adjusted *p* < 0.05 across 45 component–outcome tests; open circles indicate non-significant associations. Component categories are shown by color: recommended components, optimum-range components, and components to limit. Components to limit are reverse-coded, such that higher scores indicate lower consumption and greater adherence to PHDI recommendations.

These findings should be interpreted according to the scoring direction of each component. In the present implementation, fish/seafood and unsaturated oils were positively scored, whereas starchy vegetables, dairy, poultry, and eggs were reverse-scored, such that higher scores for these components indicate lower consumption.

### 3.6 Sensitivity analysis

In fiber-adjusted sensitivity analyses, the associations between PHDI and composite urinary phytoestrogen concentrations were attenuated but remained significant (Table 1). After additional adjustment for dietary fiber density, participants in the highest PHDI quintile had higher urinary concentrations of total phytoestrogens, total isoflavones, and total lignans compared with those in the lowest quintile. Similar positive associations were observed in the continuous PHDI models, suggesting that the observed associations were not explained solely by dietary fiber density.

In an additional sensitivity analysis excluding participants with implausible energy intake using stricter sex-specific cutoffs (*n* = 5,081), the associations between PHDI and urinary phytoestrogen outcomes were materially consistent with the primary findings (Supplementary S4).

### 3.7 Exploratory stratified analyses

Exploratory stratified analyses are presented in Supplementary Table S5. Positive associations between PHDI and total phytoestrogens and total lignans were generally observed across sex, age group, race/ethnicity, annual family income, obesity status, and cardiometabolic condition subgroups. Associations for total isoflavones were also generally positive, although estimates were weaker or less precise in some subgroups, including adults aged ≥65 years and Hispanic participants.

## 4 Discussion

In this nationally representative sample of U.S. adults, higher adherence to the PHDI was associated with higher urinary excretion of several phytoestrogen compounds. In the quintile-based analyses, urinary total isoflavones, total lignans, and total phytoestrogens increased progressively across higher PHDI quintiles, supporting a dose–response pattern across increasing levels of PHDI adherence (Table 3; Figure 1). Consistent with these findings, complementary continuous models showed that a 1-SD higher PHDI score was associated with approximately 23.4% higher total phytoestrogens, 17.4% higher total isoflavones, and 24.6% higher total lignans (Table 4). Similar associations were observed for daidzein, genistein, O-DMA, enterodiol, and enterolactone, whereas equol showed no meaningful association with PHDI. These findings suggest that greater adherence to a planetary health-oriented dietary pattern is associated with higher urinary excretion of selected diet-derived phytoestrogens at the population level.

These findings are biologically plausible and consistent with the plant-forward structure of the PHDI. Isoflavones are especially concentrated in soy-based foods, whereas lignans are derived more broadly from fiber-rich plant sources such as whole grains, seeds, fruits, vegetables, and legumes (14, 15, 25). The component-level associations shown in Figure 2 provide an important biological check on the overall findings. The strong association between soybean/soy foods and urinary isoflavones is consistent with soy foods being major sources of daidzein and genistein, while the association between whole grains and urinary lignans is consistent with the role of whole grains and other fiber-rich plant foods as lignan sources. Positive associations for fruits, non-starchy vegetables, and nuts and seeds further suggest that the urinary phytoestrogen profile reflects a broader plant-derived exposure pattern rather than a single food source.

The interpretation of reverse-scored PHDI components requires caution. Higher scores for red and processed meat, saturated and trans fats, and added sugar and fruit juice indicate lower consumption and greater adherence to PHDI recommendations (2, 3). Therefore, the positive associations observed for these components should not be interpreted as indicating that greater intake of these foods increases urinary phytoestrogen concentrations. Rather, they likely reflect the broader substitutional structure of the PHDI, whereby lower intake of foods recommended for restriction may co-occur with higher intake of plant-derived foods that contribute to urinary phytoestrogen concentrations.

The findings for lignan-related biomarkers further support the biological coherence of the observed associations (Tables 3, 4). Dietary fiber is a central feature of plant-forward dietary patterns and is closely linked to lignan metabolism through interactions with the gut microbiota. Plant lignan precursors from whole grains, seeds, fruits, vegetables, and legumes can be converted by gut microbial enzymes into the enterolignans enterodiol and enterolactone, which are subsequently absorbed, conjugated, and excreted in urine (25–28). Thus, urinary enterolignans reflect both intake of lignan-containing plant foods and host–microbial metabolic processing. In fiber-adjusted sensitivity analyses, the associations between PHDI and total phytoestrogens, total isoflavones, and total lignans were attenuated but remained significant (Table 1). These findings suggest that the observed associations were not explained solely by dietary fiber density, but may reflect a broader combination of plant-derived bioactive compounds, dietary substitution patterns, and microbiome-related metabolism captured indirectly by higher PHDI adherence.

This suggests that the observed associations were not explained solely by dietary fiber density, but may reflect a broader constellation of plant-derived bioactive compounds and dietary substitutions captured by the PHDI.

The null association for equol provides an important counterpoint to the positive findings for daidzein and other isoflavones (Tables 3, 4). Equol is not consumed directly in substantial amounts; rather, it is produced through gut microbial conversion of daidzein and depends strongly on the equol-producer phenotype (16, 18, 29). Thus, urinary equol may reflect host gut microbial composition and conversion capacity as much as, or more than, dietary daidzein exposure. The absence of an association for equol, despite positive associations for daidzein, genistein, and O-DMA, is therefore biologically informative rather than contradictory. It suggests that dietary pattern scores may capture intake-related exposure to parent isoflavones and some primary metabolites, but may not fully capture microbiome-dependent biotransformation.

Importantly, these findings should not be interpreted as formal validation of urinary phytoestrogens as specific biomarkers of PHDI adherence. Phytoestrogens are derived from multiple plant foods, including soy foods, legumes, whole grains, fruits, vegetables, nuts, and seeds (14, 15). Therefore, higher urinary phytoestrogen concentrations may reflect plant-food consumption more generally, rather than adherence to the PHDI specifically. Association alone does not establish sensitivity, specificity, reproducibility, or individual-level classification performance. Urinary phytoestrogen concentrations reflect intake of phytoestrogen-containing foods, but they also reflect absorption, gut microbial metabolism, hepatic conjugation, renal excretion, urine dilution, and timing of urine collection (13–15, 30). Accordingly, these measures are best interpreted as complementary population-level indicators of plant-derived systemic exposure aligned with the plant-forward dimension of PHDI adherence, not as specific biomarkers, direct recovery biomarkers, or stand-alone measures of PHDI adherence.

These findings advance planetary health nutrition research by characterizing how PHDI adherence relates to urinary phytoestrogen concentrations at the population level. First, the dose–response patterns across PHDI quintiles (Table 3; Figure 1) provide population-level evidence that higher PHDI adherence is associated with measurable differences in exposure to selected plant-derived compounds. Second, the component-level findings (Figure 2) suggest that the urinary phytoestrogen signal is driven primarily by biologically plausible plant-derived components, particularly soybean/soy foods for isoflavones and whole grains for lignans. Third, the null finding for equol and the weaker associations for several optimum-range components underscore that no single biomarker class can capture the full complexity of the PHDI. Exploratory stratified analyses showed generally consistent positive associations across major demographic, socioeconomic, anthropometric, and clinical subgroups, although total isoflavone associations were weaker or less precise in some subgroups. These subgroup findings should be interpreted cautiously and not as definitive evidence of effect modification. Future studies should evaluate broader biomarker panels, including fatty acids, carotenoids, alkylresorcinols, metabolomic signatures, and microbiome-related metabolites, while also considering population heterogeneity in dietary exposure and metabolism (11).

Several limitations should be acknowledged. First, the cross-sectional design precludes causal inference. Therefore, these findings should be interpreted as exploratory and hypothesis-generating rather than causal. Prospective studies, controlled feeding studies, and clinical trials with repeated dietary and biomarker measurements are needed to determine whether changes in PHDI adherence lead to subsequent changes in urinary phytoestrogen concentrations. Second, dietary intake was assessed using 24-h recalls, which remain subject to random and systematic measurement error despite standardized administration (8, 31). Although averaging two recall days improves stability, it may not fully capture usual intake. Third, urinary phytoestrogens were measured in spot urine samples and may reflect recent intake more strongly than habitual dietary patterns. Although spot urine collection and the Day-1 24-h dietary recall were conducted during the same MEC visit, the exact temporal alignment between individual food consumption events and urinary phytoestrogen concentrations cannot be established. Moreover, the PHDI was calculated using the average of two dietary recalls, whereas urinary phytoestrogens were measured from a single spot urine specimen. Creatinine adjustment reduces variation due to urine dilution, but it cannot fully account for day-to-day variation in intake or excretion. Fourth, selection bias related to biomarker availability is possible because a large proportion of eligible adults were excluded, primarily due to missing urinary phytoestrogen measurements. Included and excluded eligible participants differed in annual family income, alcohol drinking, moderate physical activity, and fiber intake density, and urinary phytoestrogen concentrations could not be compared for most excluded participants by definition. Therefore, the final analytic sample may not fully represent all eligible NHANES adults. Fifth, urinary phytoestrogen concentrations are influenced by biological processes, including absorption, gut microbial metabolism, hepatic conjugation, and renal handling (14, 15). These factors may contribute to inter-individual variation in urinary concentrations independent of dietary intake. Sixth, equol-producer phenotype and gut microbiome composition were not available in NHANES 2003–2010. This limited our ability to characterize microbial contributions to interindividual variation, particularly for equol. Seventh, residual confounding by unmeasured dietary, socioeconomic, behavioral, food-processing, or microbiome-related factors remains possible. Eighth, we did not compare the PHDI with alternative dietary quality indices, such as the Healthy Eating Index or plant-based diet indices (32, 33). Therefore, we could not determine whether urinary phytoestrogens are more strongly associated with the PHDI than with other healthy or plant-forward dietary patterns. Finally, because we did not evaluate predictive discrimination, sensitivity, specificity, or reproducibility, these findings should not be interpreted as demonstrating that urinary phytoestrogens can classify individual PHDI adherence.

## 5 Conclusion

In conclusion, higher PHDI adherence was associated with higher urinary concentrations of total phytoestrogens, total isoflavones, and total lignans among U.S. adults. Component-level patterns were consistent with dietary sources of these compounds, particularly soybean/soy foods for isoflavones and whole grains and other fiber-rich plant foods for lignans. These associations were attenuated but remained evident after additional adjustment for dietary fiber density, suggesting that they were not explained solely by fiber intake. The absence of an association for equol further suggests that microbiota-dependent metabolites may reflect host-specific biological processes beyond dietary pattern adherence alone.

These cross-sectional findings support a biological coherence between planetary health-oriented dietary patterns and systemic exposure to selected plant-derived bioactive compounds. However, urinary phytoestrogens should be interpreted as complementary population-level exposure indicators rather than validated or specific biomarkers of PHDI adherence. Longitudinal studies, controlled feeding studies, and clinical trials with repeated dietary assessment, repeated biospecimen collection, broader biomarker panels, and microbiome data are warranted to clarify the temporal and host-specific determinants of phytoestrogen exposure under plant-forward dietary patterns.

## Data Availability

The datasets presented in this study can be found in online repositories. The names of the repository/repositories and accession number(s) can be found below: https://www.cdc.gov/nchs/nhanes/index.htm.
