# Supplementary material for: Adherence to the planetary health diet index and urinary phytoestrogen excretion: a nationally representative cross-sectional study of US adults
Source: Front Nutr. 2026 Oct 1;13:1915004. doi: 10.3389/fnut.2026.1915004 (PMC13668565; doi:10.3389/fnut.2026.1915004)
Supplement: Supplementary file 1 [file Table_1.docx]

**Supplementary Figures**

Supplementary Figure S1. Flowchart of the study population

Supplementary Figure S2. Restricted cubic spline analyses of the associations between PHDI and composite urinary phytoestrogen concentrations

**Supplementary Tables**

Supplementary Table S1. Comparison of included and excluded eligible participants

Supplementary Table S2. Distributional characteristics of the PHDI and urinary phytoestrogen concentrations

Supplementary Table S3. Baseline PHDI component scores according to PHDI quintiles

Supplementary Table S4. Sensitivity analysis of PHDI–urinary phytoestrogen associations using stricter sex-specific energy intake cutoffs (n = 5,081)

Supplementary Table S5. Exploratory stratified analyses of the associations between continuous PHDI score and composite urinary phytoestrogen concentrations

Supplementary Figure S1. Flowchart of the study population

Supplementary Figure S2. Restricted cubic spline analyses of the associations between PHDI and composite urinary phytoestrogen concentrations

Solid lines indicate estimated associations, and shaded areas indicate 95% confidence intervals. Models were adjusted for age, sex, race/ethnicity, annual family income, smoking status, alcohol use, moderate physical activity, and total energy intake. P-values for overall association and non-linearity are shown within each panel.

| Supplementary Table S1. Comparison of included and excluded eligible participants | | | |
| --- | --- | --- | --- |
| Characteristics | Included | Excluded | p-value |
| Age, years, mean (SD) | 50.19 (18.10) | 50.49 (18.40) | 0.3073 |
| Sex, n (%) |  |  | 0.3434 |
| Men | 2746 (28.49) | 6891 (71.51) |  |
| Women | 2670 (27.88) | 6907 (72.12) |  |
| Race/ethnicity, n (%) |  |  | 0.6962 |
| Non-Hispanic White | 2738 (28.51) | 6867 (71.49) |  |
| Non-Hispanic Black | 1082 (28.08) | 2771 (71.92) |  |
| Hispanic | 1375 (27.87) | 3559 (72.13) |  |
| Others | 221 (26.89) | 601 (73.11) |  |
| Annual family income, n (%) |  |  | 0.0391 |
| < $20,000 | 1329 (28.07) | 3406 (71.93) |  |
| ≥$20,000 | 4087 (29.65) | 9698 (70.35) |  |
| Cigarette smoking, n (%) |  |  | 0.3319 |
| Current smoker | 1208 (28.24) | 3070 (71.76) |  |
| Past smoker | 1431 (28.97) | 3509 (71.03) |  |
| Never smoked | 2777 (27.81) | 7210 (72.19) |  |
| Alcohol drinking, n (%) |  |  |  |
| Yes | 3922 (30.71) | 8850 (69.29) | 0.0082 |
| No | 1494 (28.71) | 3709 (71.29) |  |
| Moderate activity, n (%) |  |  | 0.0036 |
| Yes | 2454 (29.42) | 5888 (70.58) |  |
| No | 2887 (27.29) | 7692 (72.71) |  |
| Unable | 75 (25.77) | 216 (74.23) |  |
| Obesity (BMI≥30kg/m^2^), n (%) |  |  | 0.1286 |
| Yes | 1999 (29.23) | 4839 (70.77) |  |
| No | 3417 (28.20) | 8702 (71.80) |  |
| Comorbidities |  |  |  |
| Diabetes | 731 (27.68) | 1910 (72.32) | 0.5314 |
| Hypertension | 2072 (28.90) | 5098 (71.10) | 0.7637 |
| Dyslipidemia | 2096 (28.23) | 5330 (71.77) | 0.9272 |
| Dietary factor, mean (SD) |  |  |  |
| Total energy intake(kcal/d) | 2057.49 (832.19) | 2057.49 (841.90) | 0.1016 |
| Fiber intake density (g/1000kcal) | 8.11 (3.77) | 8.25 (3.83) | 0.0229 |
| The included group comprised the final analytic sample (n = 5,416). The excluded group comprised eligible participants excluded because of missing urinary phytoestrogen measurements (n = 13,126) or missing covariates (n = 672), totaling 13,798 participants. P-values were calculated using t-tests for continuous variables and chi-square tests for categorical variables. | | | |

| Supplementary Table S2. Distributional characteristics of the PHDI and urinary phytoestrogen concentrations | | |
| --- | --- | --- |
| Variable | Mean (SD) | Median (IQR) |
| PHDI total score | 59.83 (14.22) | 58.86 (49.55–69.16) |
| ***Composite measures (ng/mg creatinine)*** | | |
| Total phytoestrogens | 16.87 (45.79) | 7.11 (3.10–14.95) |
| Total isoflavones | 6.94 (31.24) | 0.95 (0.40–3.22) |
| Total lignans | 9.93 (31.93) | 4.47 (1.58–10.08) |
| ***Individual analytes (ng/mg creatinine)*** | | |
| Daidzein | 3.95 (19.48) | 0.52 (0.19–1.94) |
| Genistein | 1.69 (8.35) | 0.23 (0.09–0.81) |
| O-DMA | 0.97 (7.56) | 0.03 (0.01–0.19) |
| Equol | 0.60 (5.63) | 0.06 (0.03–0.13) |
| Enterodiol | 1.55 (9.18) | 0.42 (0.16–1.03) |
| Enterolactone | 8.40 (26.99) | 3.59 (0.97–8.87) |
| O-DMA: O-Desmethylangolensin | | |

| Supplementary Table S3. Baseline PHDI component scores according to PHDI quintiles | | | | | | | |
| --- | --- | --- | --- | --- | --- | --- | --- |
| PHDI Component | Total | PHDI quintiles | | | | | P-value |
|  |  | Q1 | Q2 | Q3 | Q4 | Q5 |  |
| Recommended components | |  |  |  |  |  |  |
| Whole grains | 2.34(0.04) | 1.08(0.06) | 1.53(0.06) | 2.27(0.08) | 2.76(0.09) | 4.04(0.10) | <.0001 |
| Whole fruits | 3.96(0.05) | 1.74(0.08) | 2.80(0.10) | 3.65(0.11) | 5.03(0.12) | 6.60(0.11) | <.0001 |
| Non-starchy vegetables | 4.66(0.05) | 3.07(0.08) | 3.96(0.09) | 4.55(0.10) | 5.26(0.10) | 6.44(0.10) | <.0001 |
| Nuts and seeds | 2.03(0.04) | 0.69(0.05) | 1.12(0.07) | 1.58(0.09) | 2.54(0.11) | 4.20(0.13) | <.0001 |
| Non-soy legumes | 0.54(0.02) | 0.47(0.03) | 0.53(0.03) | 0.55(0.04) | 0.53(0.04) | 0.63(0.04) | 0.0275 |
| Soybean / soy foods | 0.14(0.01) | 0.05(0.01) | 0.06(0.01) | 0.14(0.02) | 0.13(0.02) | 0.33(0.03) | <.0001 |
| Optimum-range components | |  |  |  |  |  |  |
| Fish / seafood | 2.45(0.06) | 0.47(0.06) | 1.05(0.09) | 1.85(0.11) | 3.32(0.14) | 5.52(0.14) | <.0001 |
| Eggs | 8.27(0.04) | 7.15(0.11) | 8.02(0.09) | 8.56(0.07) | 8.78(0.06) | 8.86(0.06) | <.0001 |
| Dairy | 7.98(0.04) | 7.06(0.10) | 8.03(0.08) | 8.02(0.08) | 8.26(0.08) | 8.52(0.07) | <.0001 |
| Poultry | 7.31(0.05) | 6.17(0.13) | 7.02(0.12) | 7.51(0.11) | 7.71(0.11) | 8.14(0.10) | <.0001 |
| Starchy vegetables | 8.34(0.04) | 6.96(0.11) | 8.15(0.09) | 8.63(0.08) | 8.81(0.08) | 9.13(0.06) | <.0001 |
| Unsaturated oils | 3.03(0.04) | 1.88(0.06) | 2.32(0.07) | 2.84(0.08) | 3.43(0.08) | 4.66(0.09) | <.0001 |
| Components to limit (reverse-coded) | |  |  |  |  |  |  |
| Red and processed meat | 4.63(0.05) | 2.27(0.10) | 3.83(0.11) | 4.61(0.12) | 5.57(0.12) | 6.84(0.11) | <.0001 |
| Saturated and trans fats | 0.46(0.02) | 0.14(0.02) | 0.17(0.02) | 0.35(0.04) | 0.50(0.04) | 1.17(0.07) | <.0001 |
| Added sugar and fruit juice | 3.70(0.05) | 2.00(0.09) | 2.95(0.11) | 3.74(0.11) | 4.26(0.12) | 5.53(0.11) | <.0001 |
| Values are presented as mean (SE) of PHDI component scores. Higher component scores indicate greater adherence to the PHDI recommendation. For components to limit, higher scores indicate lower consumption because these components are reverse-coded. P-values were calculated for trend across PHDI quintiles. | | | | | | | |

| Supplementary Table S4. Sensitivity analysis of PHDI–urinary phytoestrogen associations using stricter sex-specific energy intake cutoffs (n = 5,081) | | | | | | | | | | | | | |
| --- | --- | --- | --- | --- | --- | --- | --- | --- | --- | --- | --- | --- | --- |
| Phytoestrogen | PHDI quintiles (Q1 as reference), β (95% CI) p-value | | | | | | | | | PHDI per 1-SD increase | | |  |
|  | Q2 | | Q3 | | Q4 | | Q5 | | β (95% CI) | | p-value |  |  |
|  | β (95% CI) | p-value | β (95% CI) | p-value | β (95% CI) | p-value | β (95% CI) | p-value |  |  |  |  |  |
| Composite measures |  |  |  |  |  |  |  |  |  | |  |  |  |
| Total phytoestrogens | 0.12  (-0.04–0.28) | 0.1475 | 0.29  (0.12–0.45) | 0.0016 | 0.45  (0.31–0.60) | <.0001 | 0.82  (0.66–0.97) | <.0001 | 0.21  (0.18–0.25) | | <0.001 |  |  |
| Total Isoflavons | 0.10  (-0.06–0.27) | 0.2194 | 0.00  (-0.16–0.16) | 0.9969 | 0.29  (0.13–0.46) | 0.0011 | 0.59  (0.40–0.78) | <.0001 | 0.16  (0.12–0.21) | | <0.001 |  |  |
| Total lignans | 0.13  (-0.07–0.33) | 0.2186 | 0.41  (0.18–0.64) | 0.0009 | 0.48  (0.26–0.70) | <.0001 | 0.84  (0.65–1.04) | <.0001 | 0.22  (0.18–0.26) | | <0.001 |  |  |
| Individual analytes |  |  |  |  |  |  |  |  |  | |  |  |  |
| Daidzein | 0.09  (-0.08–0.27) | 0.3169 | 0.02  (-0.17–0.21) | 0.8319 | 0.29  (0.09–0.49) | 0.0056 | 0.61  (0.41–0.80) | <0.0001 | 0.17  (0.12–0.21) | | <0.001 |  |  |
| Genistein | 0.14  (-0.02–0.31) | 0.0989 | 0.00  (-0.17–0.17) | 0.9921 | 0.33  (0.14–0.52) | 0.0013 | 0.62  (0.42–0.82) | <0.0001 | 0.18  (0.13–0.23) | | <0.001 |  |  |
| O-DMA | 0.07  (-0.18–0.32) | 0.5888 | 0.05  (-0.18–0.29) | 0.6607 | 0.38  (0.15–0.61) | 0.0018 | 0.70  (0.39–1.01) | <0.0001 | 0.20  (0.13–0.26) | | <0.001 |  |  |
| Equol | 0.02  (-0.10–0.15) | 0.7325 | -0.02  (-0.18–0.14) | 0.8033 | 0.04  (-0.12–0.20) | 0.6284 | 0.07  (-0.07–0.20) | 0.3337 | 0.02  (-0.01–0.05) | | 0.2529 |  |  |
| Enterodiol | 0.22  (0.04–0.40) | 0.0196 | 0.39  (0.19–0.60) | 0.0005 | 0.41  (0.21–0.61) | 0.0002 | 0.81  (0.62–1.01) | <0.0001 | 0.21  (0.16–0.25) | | <0.001 |  |  |
| Enterolactone | 0.09  (-0.13–0.30) | 0.4337 | 0.41  (0.16–0.66) | 0.0025 | 0.50  (0.28–0.72) | <0.0001 | 0.80  (0.57–1.03) | <0.0001 | 0.21  (0.17–0.26) | | <0.001 |  |  |
| Values are β coefficients and 95% confidence intervals from survey-weighted linear regression models using natural log-transformed, creatinine-standardized urinary phytoestrogen concentrations. This sensitivity analysis excluded participants with implausible mean daily energy intake using stricter sex-specific cutoffs: <600 or >3,500 kcal/day for women and <800 or >4,200 kcal/day for men. Mean daily energy intake was calculated from the two 24-hour dietary recalls. Models were adjusted for age, sex, race/ethnicity, annual family income, smoking status, alcohol use, moderate physical activity, and total energy intake, consistent with the primary Model 2. PHDI quintile analyses used Q1 as the reference group. Continuous PHDI analyses were scaled per 1-SD higher PHDI score. | | | | | | | | | | | | |  |
| Supplementary Table S5. Exploratory stratified analyses of the associations between continuous PHDI score and composite urinary phytoestrogen concentrations | | | | | | | | | | | | |  |

| Stratification  variable | Subgroup | n | Total phytoestrogens | | Total isoflavones | | Total lignans | |
| --- | --- | --- | --- | --- | --- | --- | --- | --- |
|  |  |  | β (95% CI) | p-value | β (95% CI) | p-value | β (95% CI) | p-value |
| Sex | Male | 2746 | 0.20 (0.15–0.25) | <0.001 | 0.11 (0.07–0.16) | <0.001 | 0.22 (0.14–0.29) | <0.001 |
|  | Female | 2670 | 0.22 (0.17–0.27) | <0.001 | 0.21 (0.13–0.29) | <0.001 | 0.21 (0.16–0.27) | <0.001 |
| Age group | 20–44 years | 2245 | 0.24 (0.19–0.29) | <0.001 | 0.19 (0.12–0.25) | <0.001 | 0.27 (0.21–0.33) | <0.001 |
|  | 45–64 years | 1786 | 0.23 (0.18–0.28) | <0.001 | 0.19 (0.13–0.26) | <0.001 | 0.19 (0.12–0.25) | <0.001 |
|  | ≥65 years | 1385 | 0.13 (0.05–0.21) | 0.0016 | 0.03 (-0.06–0.12) | 0.5258 | 0.19 (0.09–0.28) | 0.0003 |
| Race/ethnicity | Non-Hispanic White | 2738 | 0.23 (0.19–0.27) | <0.001 | 0.18 (0.13–0.24) | <0.001 | 0.23 (0.19–0.28) | <0.001 |
|  | Non-Hispanic Black | 1082 | 0.16 (0.09–0.23) | <0.001 | 0.10 (0.00–0.20) | 0.0509 | 0.14 (0.05–0.24) | 0.0056 |
|  | Hispanic | 1375 | 0.14 (0.06–0.21) | 0.0009 | 0.04 (-0.08–0.15) | 0.5428 | 0.19 (0.11–0.27) | <0.001 |
|  | Other | 221 | 0.23 (0.00–0.47) | 0.0606 | 0.23 (-0.06–0.53) | 0.1262 | 0.24 (0.00–0.48) | 0.0535 |
| Income | Low | 1329 | 0.21 (0.14–0.28) | <0.001 | 0.10 (-0.01–0.21) | 0.0742 | 0.25 (0.17–0.34) | <0.001 |
|  | High | 4087 | 0.21 (0.18–0.25) | <0.001 | 0.17 (0.13–0.22) | <0.001 | 0.21 (0.16–0.26) | <0.001 |
| Obesity status | No | 3417 | 0.21 (0.18–0.25) | <0.001 | 0.18 (0.13–0.23) | <0.001 | 0.20 (0.16–0.25) | <0.001 |
|  | Yes | 1999 | 0.17 (0.12–0.21) | <0.001 | 0.11 (0.05–0.17) | 0.0016 | 0.19 (0.13–0.26) | <0.001 |
| Cardiometabolic condition | No |  | 0.22 (0.16–0.29) | <0.001 | 0.18 (0.11–0.26) | <0.001 | 0.24 (0.16–0.31) | <0.001 |
|  | Yes |  | 0.21 (0.16–0.26) | <0.001 | 0.15 (0.09–0.22) | <0.001 | 0.20 (0.15–0.26) | <0.001 |
| Stratified analyses were exploratory and restricted to composite urinary phytoestrogen outcomes. Values are β coefficients and 95% confidence intervals from survey-weighted linear regression models using natural log-transformed urinary phytoestrogen concentrations. PHDI was modeled continuously per 1-SD higher score. Models were adjusted for age, sex, race/ethnicity, annual family income, smoking status, alcohol use, moderate physical activity, and total energy intake, except for the stratification variable. Annual family income was categorized as < $20,000 and ≥ $20,000. Obesity was defined as body mass index ≥30 kg/m². Any cardiometabolic condition was defined as the presence of diabetes, hypertension, or dyslipidemia. | | | | | | | | |
